# Effects of Dietary Quercetin Supplementation on Growth Performance, Serum Health Indices, and Whole-Blood Gene Expression in Weaned Piglets

**DOI:** 10.3390/ani16152429

**Published:** 2026-08-06

**Authors:** Yizhuo Li, Linsen Shao, Xuancheng Guan, Liyuan Xie, Da Qiao, Jing Chen, Xianjun Liu

**Affiliations:** College of Animal Science and Veterinary Medicine, Shenyang Agricultural University, Shenyang 110866, China; 2025220620@stu.syau.edu.cn (Y.L.); 13667383305@163.com (L.S.); 15764380107@163.com (X.G.); 2025220609@stu.syau.edu.cn (L.X.); 2025240808@stu.syau.edu.cn (D.Q.)

**Keywords:** quercetin, weaned piglets, growth performance, antioxidant status, inflammatory biomarkers, gene expression

## Abstract

This study evaluated dietary quercetin supplementation to investigate the effects of increasing inclusion levels on growth performance, diarrhea, serum health-related indicators, and whole-blood gene expression in weaned piglets. In a 28-day feeding trial, 128 piglets received diets formulated to contain 0, 100, 200, or 300 mg/kg quercetin (four pens of eight piglets per treatment; pen as the experimental unit). Final body weight and average daily gain were higher in the 200 and 300 mg/kg groups than in the control, and the 300 mg/kg group had the highest gain-to-feed ratio (G:F) (*p* < 0.05). Diarrhea rate declined linearly (*p* < 0.001); the rate in the 300 mg/kg group was lower than in the control and 100 mg/kg groups but did not differ from the 200 mg/kg group. Quercetin reduced serum malondialdehyde, pro-inflammatory cytokines, urea nitrogen, and aspartate aminotransferase, while increasing total protein, albumin, total antioxidant capacity, several antioxidant enzyme activities, and interleukin-10 (*p* < 0.05). Serum diamine oxidase activity and D-lactate concentration also declined linearly (*p* < 0.001). Whole-blood expression of *PTGS2* decreased; *HMOX1*, *NFE2L2*, and *NQO1* increased; and *SOD1* was unchanged. Overall, within the tested range, increasing dietary quercetin improved growth and feed efficiency, reduced diarrhea, and was accompanied by coordinated changes in serum health-related indicators and whole-blood transcripts.

## Simple Summary

Weaning can impair piglet growth and digestive health and disturb antioxidant and immune balance. Quercetin may help piglets during weaning by supporting antioxidant defenses and moderating inflammation. The objective of this study was to determine whether increasing dietary quercetin could improve growth and feed efficiency, reduce diarrhea, and support health-related responses in weaned piglets. Supplementation improved growth and feed efficiency, reduced diarrhea, and was associated with coordinated changes in antioxidant status, inflammatory responses, serum indicators related to intestinal permeability, and selected whole-blood gene expression. Overall, quercetin may support piglet performance and physiological status after weaning.

## 1. Introduction

Weaning requires young pigs to adapt rapidly to a new diet, unfamiliar surroundings, and social regrouping. This transition is commonly accompanied by reduced feed intake and growth, disturbed redox and immune balance, altered intestinal function, and a greater risk of diarrhea [1,2]. Because these responses occur in parallel, assessment across several physiological domains is more informative than reliance on a single outcome. Recent nursery-pig studies have likewise reported concurrent variation in performance, immunoglobulins, antioxidant measures, and blood indicators related to intestinal permeability [3].

Phytogenic feed additives are being explored as nutritional tools that may reduce dependence on routine antibiotics in animal production. Phenolic compounds and flavonoids are particularly relevant because they have been linked with changes in oxidative status, immune responsiveness, and gastrointestinal function [4,5].

Quercetin is a widely studied flavonol in pig nutrition, although reported responses differ with age, diet composition, and experimental challenge. In newly weaned piglets, quercetin has been associated with glutathione-related responses and indices of small-intestinal function [6]. Other studies in nursery piglets or experimental challenge models have described changes in growth, diarrhea, immune variables, or oxidative-stress-related outcomes [7,8,9].

The systemic availability of dietary quercetin depends on its chemical form, intestinal metabolism, and the dietary matrix. In pigs, unchanged free quercetin shows limited systemic availability after oral administration, whereas conjugated and methylated metabolites account for a larger proportion of circulating quercetin-derived compounds; part of this conjugation may occur in the intestinal wall [10]. Intestinal absorption also varies with the glycoside moiety and other dietary factors [11]. In addition, quercetin and other dietary polyphenols undergo extensive gastrointestinal and microbial biotransformation before and after absorption, which may alter their subsequent biological activity [12,13]. These pharmacokinetic features should be considered when interpreting responses to graded dietary quercetin supplementation.

Studies of diverse phytogenic preparations have reported changes in growth, antioxidant status, immune measures, intestinal morphology, and microbial traits in weaned pigs [14,15,16,17,18,19,20]. Direct cross-study comparison is difficult because botanical composition, inclusion level, piglet age, feeding duration, and challenge status vary substantially. Results obtained with complex preparations should therefore not be extrapolated directly to purified quercetin. Here, commercially sourced quercetin was evaluated in a graded-inclusion trial using the pen as the experimental unit. We assessed growth, diarrhea occurrence, and serum biochemical, redox, immune, inflammatory, and permeability-related outcomes. Selected whole-blood transcripts were included as systemic molecular observations and were not used to establish activation of a tissue-specific pathway.

## 2. Materials and Methods

### 2.1. Ethics Statement

All procedures involving animals were approved by the Experimental Animal Welfare and Ethics Committee of Shenyang Agricultural University (protocol SNLL25091801; approval date: 18 September 2025). The study was conducted in accordance with the institution’s animal-care requirements.

### 2.2. Quercetin Source, Diet Preparation, and Storage

Purified quercetin was obtained commercially as a dry powder (supplier-reported high-performance liquid chromatography (HPLC) purity ≥99.5%; catalog no. TZ-E005; Xi’an Tianzi Biotechnology Co., Ltd., Xi’an, China). For each quercetin-supplemented treatment, the required amount of quercetin was first blended with a small quantity of the basal diet to prepare a premix and was then incorporated into the complete diet by stepwise dilution to promote uniform distribution. All four experimental diets were prepared once weekly throughout the 28-day feeding trial, resulting in four preparation rounds in total. After preparation, each diet was stored in sealed, opaque polypropylene bags in a dry, well-ventilated feed-storage room at an ambient temperature of 20–25 °C. The room temperature was checked daily using a thermometer, and the diets were protected from direct sunlight until use. The actual quercetin concentrations, recovery, homogeneity, and stability in the finished diets were not independently verified after feed preparation or during storage. Premixing and stepwise dilution were used to promote uniform distribution, whereas weekly preparation, storage in sealed opaque polypropylene bags, and protection from direct sunlight were used to minimize the potential degradation of quercetin during the feeding trial. This animal study did not involve the extraction of plant material or in-house compositional characterization.

### 2.3. Animals, Experimental Design, Diets, and Management

All 128 healthy Duroc × Landrace × Large White crossbred piglets were weaned on the same day at 28 days of age and then acclimated to the housing and feeding conditions for 4 days. During the acclimation period, all piglets were fed the basal diet without supplemental quercetin, identical to the diet subsequently provided to the control group. At 32 days of age, the piglets had an initial body weight of 10.28 ± 0.04 kg (mean ± SEM). Before treatment allocation, piglets were stratified by sex and initial body weight. Within these strata, restricted randomization was used to assign piglets to treatments and pens, while littermates were distributed as evenly as possible across treatments and pens. This procedure achieved comparable initial body weights among treatments, and each pen contained four barrows and four gilts. Initial body weight, sex, and litter origin were used only to balance animal allocation and were not treated as formal blocks or included as blocking factors in the statistical model. Accordingly, the study was analyzed as a completely randomized design, with dietary treatment as the sole fixed factor and the pen as the experimental unit. Each treatment comprised four pens of eight piglets, and the feeding trial lasted 28 days.

The control treatment (CON) received the basal diet without supplemental quercetin. Diets Q100, Q200, and Q300 were formulated to contain 100, 200, and 300 mg/kg quercetin, respectively. These concentrations represent the formulated dietary inclusion levels and were not analytically verified in the finished diets after feed preparation. Quercetin replaced an equal mass of ground peanut hulls to maintain the dietary formulation. Diets were formulated to satisfy the nutrient recommendations of the National Research Council (NRC) for weaned pigs [21]. The ingredient composition and nutrient profile of the experimental diets are presented in Table 1. Piglets were housed in a single environmentally controlled nursery room at 28 ± 1 °C during the first week and 25 °C thereafter, with mechanical ventilation and a 12 h light/12 h dark schedule. The 16 pens, each measuring 1.8 m × 1.5 m, had plastic slatted floors, and housed four barrows and four gilts. Pen locations were randomly assigned, and the four replicate pens of each treatment were spatially interspersed throughout the room to minimize potential positional effects. Piglets had ad libitum access to feed and water throughout the trial. Feeders were checked and replenished at 08:00 and 16:00 daily to maintain continuous feed availability. Health was checked every day; no piglets were removed because of disease or mortality.

### 2.4. Growth Performance, Feed Intake, and Diarrhea Assessment

Initial body weight (IBW) was recorded on the morning of day 0, when the piglets were 32 days old and had completed the 4-day acclimation period. To minimize variation associated with gastrointestinal contents, feed was withdrawn for 12 h overnight before weighing, while water remained available ad libitum. All piglets were weighed individually between 08:00 and 09:00 using an electronic livestock platform scale (TCS/SCS-S series; Shanghai Tianhe Electronic Co., Ltd., Shanghai, China), before the morning feeding and initiation of the dietary treatments. At the end of day 28, feed was again withdrawn for 12 h overnight, while water remained available ad libitum, and final body weight (FBW) was recorded on the morning of day 29 using the same weighing procedure. At both weighing occasions, the piglets were weighed in the same pen order and within-pen animal sequence to maintain procedural consistency. Average daily gain (ADG, g/pig/day) was calculated as follows:

(1) $$\mathrm{ADG}\;=\;\frac{(\mathrm{FBW}\;-\;\mathrm{IBW})\;\times \;1000}{28}$$

The amounts of feed offered and feed remaining were weighed and recorded separately for each pen throughout the trial. Pen-level feed disappearance was calculated as the cumulative amount of feed offered minus the cumulative amount of feed remaining. Average daily feed intake (ADFI, g/pig/day) was calculated as follows:

(2) $$\mathrm{ADFI}\;=\;\frac{\mathrm{total}\;\mathrm{pen}-\mathrm{level}\;\mathrm{feed}\;\mathrm{disappearance}}{\mathrm{number}\;\mathrm{of}\;\mathrm{piglets}\;\mathrm{per}\;\mathrm{pen}\;\times \;\mathrm{trial}\;\mathrm{duration}}$$

Gain-to-feed ratio (G:F) was calculated as follows:

(3) $$G:F\;=\;\frac{\mathrm{total}\;\mathrm{pen}\;\mathrm{body}-\mathrm{weight}\;\mathrm{gain}}{\mathrm{total}\;\mathrm{pen}-\mathrm{level}\;\mathrm{feed}\;\mathrm{disappearance}}$$

Fecal consistency was observed and scored once daily for each piglet throughout the 28-day feeding trial using a five-point scale: 1, severe diarrhea, characterized by watery and unformed feces with fecal-water separation; 2, moderate diarrhea, characterized by semi-liquid feces without fecal-water separation; 3, mild diarrhea, characterized by soft and partly formed feces; 4, normal, well-formed feces; and 5, hard feces or constipation. One daily score was recorded for each piglet and used to classify the corresponding pig-day as diarrheal or non-diarrheal. Scores of 1–3 were classified as diarrhea because score 3 represented mild diarrhea rather than normal fecal consistency. Although the direction of the scale differs among published systems, recent piglet studies have likewise classified an intermediate fecal-consistency score as mild diarrhea [22,23]. The same scoring criteria were applied consistently throughout the 28-day trial. Diarrhea rate was calculated as follows:

(4) $$\mathrm{Diarrhea}\;\mathrm{rate}\;(\%)=\frac{\mathrm{diarrheal}\;\mathrm{pig}-\mathrm{days}}{\mathrm{total}\;\mathrm{pig}-\mathrm{days}}\times 100$$

### 2.5. Sample Collection and Serum Assays

On day 29, after final body weight had been measured, two piglets with body weights closest to the pen mean were selected from each pen (eight piglets per treatment). Blood (approximately 10 mL) was drawn from the anterior vena cava. Serum was prepared from blood collected into tubes without anticoagulant, and BD Vacutainer K_2_EDTA tubes (Becton, Dickinson and Company, Franklin Lakes, NJ, USA) were used for whole-blood RNA analysis. The serum tubes were maintained at 4 °C for 30 min before centrifugation at 3000× *g* for 15 min at 4 °C using a Sorvall ST 8R refrigerated benchtop centrifuge (Thermo Fisher Scientific, Waltham, MA, USA). Serum aliquots were stored at −80 °C. Whole-blood samples were placed on ice immediately and transferred to −80 °C within 2 h. The two piglets sampled from each pen were treated as biological subsamples rather than independent experimental units. Their values were averaged to obtain one pen-level value for each serum and whole-blood endpoint before statistical analysis.

Commercial kits from Nanjing Jiancheng Bioengineering Institute (Nanjing, China) were used according to the manufacturers’ instructions to determine the serum biochemical, antioxidant, immune, inflammatory, and intestinal permeability-related indicators. Serum samples were labeled with coded identifiers, and the investigator performing the assays remained blinded to dietary treatment allocation until all measurements and data recording had been completed. Detailed information on the analytical endpoints, assay methods, catalog numbers, and reported units is provided in Supplementary Table S4.

### 2.6. Whole-Blood Reverse-Transcription Quantitative Polymerase Chain Reaction (RT-qPCR)

Total RNA was isolated from K_2_EDTA-anticoagulated whole blood with TRIzol reagent (catalog no. 15596026; Invitrogen, Thermo Fisher Scientific, Waltham, MA, USA), and RNA concentration and purity were assessed using a NanoDrop One microvolume UV–Vis spectrophotometer (Thermo Fisher Scientific, Waltham, MA, USA). Complementary DNA (cDNA) was synthesized using PrimeScript RT Master Mix (RR036A; Takara Bio, Beijing, China). RT-qPCR was performed using TB Green Premix Ex Taq II (RR820A; Takara Bio) on an Accurate 96 real-time PCR system (DLAB Scientific Co., Ltd., Beijing, China). Actin beta (*ACTB*) served as the reference gene, and each cDNA sample was analyzed in triplicate. Primer amplification efficiencies were assessed by standard-curve analysis before relative quantification. The efficiencies ranged from 95.7% to 98.6%, and the corresponding coefficients of determination (*R*^2^) ranged from 0.995 to 0.998. The comparable amplification efficiencies of the target genes and *ACTB* supported the use of the 2^−ΔΔCt^ method. Relative mRNA expression was calculated using the 2^−ΔΔCt^ method [24]. The mean ΔCt value of the CON group was used as the calibrator for relative expression calculations. Individual amplification efficiencies and *R*^2^ values, together with primer sequences and amplicon information, are provided in Supplementary Table S1; reverse-transcription and qPCR reaction conditions are provided in Supplementary Table S2.

### 2.7. Statistical Analysis

All statistical analyses used the pen as the experimental unit (*n* = 4 pens per dietary treatment). For serum and whole-blood endpoints, the two sampled piglets within each pen were regarded as biological subsamples, and their values were averaged to obtain one pen-level observation before statistical analysis. Results are expressed as means ± SEM. Normality of the model residuals and homogeneity of variances were assessed using the Shapiro–Wilk test and Levene’s test, respectively. Treatment effects were assessed using one-way ANOVA. Planned orthogonal polynomial contrasts were the primary analyses used to evaluate linear and quadratic dose–response patterns across the graded quercetin inclusion levels. When the overall treatment effect was significant, Tukey’s multiple-comparison test was used as a secondary analysis to identify specific pairwise differences among treatment means. Partial eta squared (*η_p_*^2^) was calculated as an estimate of effect size for the overall dietary treatment effect. For endpoints that did not meet one or both assumptions, the overall treatment effect was additionally evaluated in sensitivity analyses using Welch’s ANOVA or the Kruskal–Wallis test, as appropriate. Diarrhea-rate proportions were subjected to an arcsine square-root transformation before statistical analysis. For this endpoint, the transformed data were used for one-way ANOVA, Tukey’s multiple-comparison test, and orthogonal polynomial contrast analyses, whereas untransformed percentages are presented in Table 2 for ease of interpretation. Spearman correlation analysis and principal component analysis (PCA) were performed with OriginPro 2021 (OriginLab Corporation, Northampton, MA, USA). All univariate statistical analyses were performed using IBM SPSS Statistics 27.0 (IBM Corp., Armonk, NY, USA). Differences were considered significant at *p* < 0.05, whereas 0.05 ≤ *p* < 0.10 was considered indicative of a statistical tendency.

Exploratory correlation analysis and PCA were performed with pooled data from 16 pens. The nine selected variables were ADG, diarrhea rate, UN, MDA, GSH-Px, TNF-α, IL-10, DAO, and D-lactate; the same variables were z-score standardized for PCA. Collectively, they represented production performance, clinical response, nitrogen metabolism, redox status, inflammatory balance, and serum permeability-related indices. These multivariable procedures were descriptive and were not used to infer causal relationships or associations independent of dietary treatment. G:F was included in the primary univariate and dose–response analyses but excluded from the multivariable set to avoid duplicating growth-related information.

## 3. Results

### 3.1. Growth Performance, Feed Intake, and Diarrhea Rate

Initial body weight was comparable among treatments (*p*-treatment = 0.867; Table 2). FBW and ADG increased linearly with increasing quercetin inclusion (both *p*-linear < 0.001). Pairwise comparisons showed that FBW was higher in Q200 and Q300 than in CON and Q100, whereas ADG was higher in Q200 and Q300 than in CON and was also higher in Q300 than in Q100 (*p* < 0.05). ADFI decreased linearly with increasing quercetin inclusion (*p*-linear < 0.001). ADFI did not differ between CON and Q100 but was lower in Q200 and Q300 than in both CON and Q100; it was also lower in Q300 than in Q200 (*p* < 0.05). G:F increased linearly with increasing quercetin inclusion (*p*-linear < 0.001). G:F was higher in Q200 and Q300 than in both CON and Q100 and was also higher in Q300 than in Q200 (*p* < 0.05). Diarrhea rate declined linearly across the inclusion range (*p*-linear < 0.001). The rate was lower in Q300 than in CON and Q100 but did not differ significantly from Q200. No significant quadratic responses were detected for the variables presented in Table 2 (all *p*-quadratic > 0.05). Partial eta-squared values (*η_p_*^2^) for FBW, ADG, ADFI, G:F, and diarrhea rate ranged from 0.652 to 0.920, whereas the value for IBW was 0.056 (Table 2).

### 3.2. Serum Biochemical Parameters

Several serum biochemical endpoints differed among dietary treatments (Table 3). Albumin (ALB), total protein (TP), glucose (GLU), and high-density lipoprotein cholesterol (HDL-C) increased linearly with increasing dietary quercetin inclusion (all *p*-linear < 0.001). Pairwise comparisons showed that ALB, TP, and HDL-C were higher in all quercetin-supplemented groups than in CON, whereas GLU was higher in Q200 and Q300 than in CON. In contrast, urea nitrogen (UN), aspartate aminotransferase (AST), alanine aminotransferase (ALT), creatinine (CREA), triglycerides (TGs), total cholesterol (TC), low-density lipoprotein cholesterol (LDL-C), lactate dehydrogenase (LDH), and γ-glutamyl transferase (GGT) decreased linearly with increasing dietary quercetin inclusion (all *p*-linear < 0.001). Significant quadratic responses were detected for ALB (*p*-quadratic = 0.042), TP, UN, and AST (all *p*-quadratic < 0.001). Tendencies toward quadratic responses were observed for TC (*p*-quadratic = 0.050) and LDH (*p*-quadratic = 0.060). Total bilirubin (TBIL) showed neither a significant treatment effect nor significant linear or quadratic responses. Direct bilirubin (DBIL) decreased linearly with increasing dietary quercetin inclusion (*p*-linear = 0.027), despite the absence of a significant overall treatment effect (*p*-treatment = 0.131). Alkaline phosphatase (ALP) reached its lowest value in Q200 and was lower than in CON, Q100, and Q300 (all *p* < 0.05), whereas Q100 and Q300 did not differ from CON. ALP showed significant linear (*p*-linear = 0.009) and quadratic (*p*-quadratic < 0.001) components. Creatine kinase (CK) differed among treatments (*p*-treatment < 0.001) and displayed a non-monotonic pattern but showed neither a significant linear nor quadratic response. Linear and quadratic contrast results for all measured endpoints are provided in Supplementary Table S3F.

### 3.3. Serum Antioxidant Parameters

Dietary quercetin altered serum antioxidant measurements (Table 4). Relative to CON, all quercetin-supplemented groups had higher values of total antioxidant capacity (T-AOC), total superoxide dismutase (T-SOD), glutathione reductase activity coefficient (GRAC), glutathione peroxidase (GSH-Px), and catalase (CAT), and lower values of peroxidase (POD) and malondialdehyde (MDA) (*p* < 0.05). For both T-AOC and GRAC, Q100 and Q200 did not differ, whereas Q300 was higher than both treatments (*p* < 0.05). With increasing dietary quercetin inclusion, T-AOC, T-SOD, GRAC, GSH-Px, and CAT increased linearly, whereas POD and MDA decreased linearly (all *p*-linear < 0.001). Significant quadratic responses were detected for T-AOC, T-SOD, MDA, and GSH-Px (all *p*-quadratic < 0.001). CAT showed a tendency toward a quadratic response (*p*-quadratic = 0.065), whereas POD and GRAC showed no quadratic response (*p*-quadratic = 0.113 and 0.723, respectively; Table 4). Because the model residuals for POD deviated from normality, a Kruskal–Wallis sensitivity analysis was performed. The overall treatment effect remained significant (*p* = 0.004).

### 3.4. Serum Immune and Inflammatory Parameters

Across the graded quercetin inclusion levels, serum IgA, IgG, IgM, and IL-10 increased linearly, whereas IL-1β, TNF-α, and IL-6 decreased linearly (*p*-linear = 0.011 for IgM and *p*-linear < 0.001 for all other variables listed above; Table 5). Pairwise comparisons showed that IgA was higher in all quercetin-supplemented groups than in CON; IgG and IgM were higher in Q100 and Q300 than in CON, whereas Q200 did not differ from CON. IL-1β, TNF-α, and IL-6 were lower, and IL-10 was higher, in all supplemented groups than in CON. Significant quadratic responses were detected for IL-1β, IL-10, and Hs-CRP (*p*-quadratic < 0.001, 0.004, and < 0.001, respectively). Hs-CRP also showed a significant linear contrast (*p*-linear = 0.020), although the group means were non-monotonic and highest in Q100. LZM was unaffected by dietary treatment. Because the model residuals for IgG deviated from normality and the assumption of homogeneity of variances was not met for IgG and Hs-CRP, sensitivity analyses were performed. The overall treatment effects remained significant for IgG (Kruskal–Wallis, *p* = 0.003) and Hs-CRP (Welch’s ANOVA, *p* = 0.018).

### 3.5. Serum Diamine Oxidase Activity and D-Lactate Concentration

**Figure 1:**
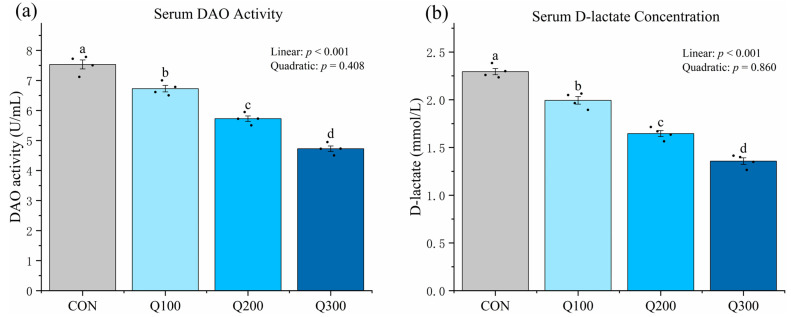
Serum DAO activity and D-lactate concentration in weaned piglets receiving graded dietary quercetin. (**a**) DAO activity; (**b**) D-lactate concentration. Individual points represent pen-level observations, each calculated as the mean of the two sampled piglets within a pen; bars and error bars represent treatment means ± SEM (*n* = 4 pens per treatment). Within each panel, bars without a common lowercase letter differ according to Tukey’s multiple-comparison test (*p* < 0.05). Both variables decreased linearly with increasing dietary quercetin inclusion (both *p*-linear < 0.001). Linear and quadratic contrast *p*-values are displayed within each panel.

Serum diamine oxidase (DAO) activity and D-lactate concentration were lower in all quercetin treatments than in CON (Figure 1). Both DAO activity and D-lactate concentration decreased linearly with increasing dietary quercetin inclusion (both *p*-linear < 0.001), whereas their quadratic responses were not significant (DAO, *p*-quadratic = 0.408; D-lactate, *p*-quadratic = 0.860).

### 3.6. Whole-Blood Relative mRNA Expression

**Figure 2:**
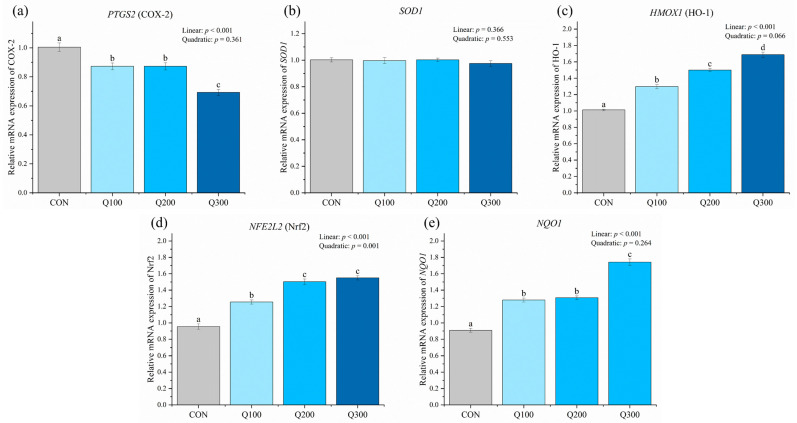
Effects of graded dietary quercetin on whole-blood mRNA expression in weaned piglets. (**a**) *PTGS2* (COX-2); (**b**) *SOD1*; (**c**) *HMOX1* (HO-1); (**d**) *NFE2L2* (Nrf2); and (**e**) *NQO1*. Relative mRNA expression was calculated by the 2^−ΔΔCt^ method with *ACTB* as the reference gene. Primer amplification efficiencies and *R*^2^ values are provided in Supplementary Table S1. Values are means ± SEM (*n* = 4 pens per treatment) after averaging the two sampled piglets within each pen. Within each panel, bars without a common lowercase letter differ according to Tukey’s multiple-comparison test (*p* < 0.05). Linear and quadratic contrast *p*-values are displayed within each panel.

*PTGS2* expression decreased linearly, whereas the expression of *HMOX1*, *NFE2L2*, and *NQO1* increased linearly with increasing quercetin inclusion (all *p*-linear < 0.001; Figure 2). *SOD1* showed no significant treatment effect or linear or quadratic response (*p*-treatment = 0.685, *p*-linear = 0.366, and *p*-quadratic = 0.553). *HMOX1* showed a tendency toward a quadratic response (*p*-quadratic = 0.066), whereas *NFE2L2* showed a significant quadratic response (*p*-quadratic = 0.001); the quadratic responses of *PTGS2* and *NQO1* were not significant (*p*-quadratic = 0.361 and 0.264, respectively). Pairwise comparisons showed that *PTGS2* expression was lower in Q100 and Q200 than in CON and was lowest in Q300. *HMOX1* expression increased progressively from CON to Q300, and all four groups differed from one another. *NFE2L2* expression was higher in Q100 than in CON and was higher in Q200 and Q300 than in Q100, with no difference between Q200 and Q300. *NQO1* expression was higher in Q100 and Q200 than in CON, did not differ between Q100 and Q200, and was highest in Q300. All pairwise differences described above were significant (*p* < 0.05).

### 3.7. Correlation Analysis and Principal Component Analysis

**Figure 3:**
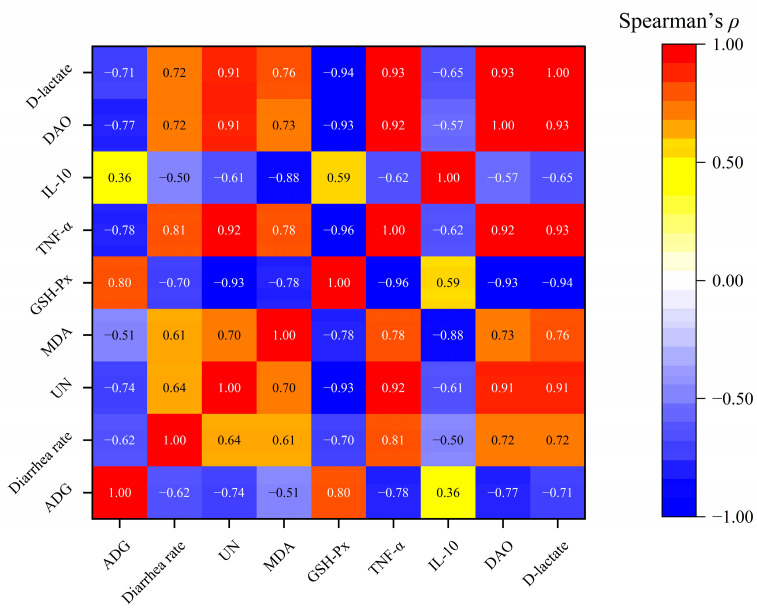
Exploratory Spearman correlation matrix for nine variables measured in weaned piglets: ADG, diarrhea rate, UN, MDA, GSH-Px, TNF-α, IL-10, DAO, and D-lactate. Coefficients were calculated from pooled pen-level observations (*n* = 16). Cells show Spearman’s *ρ*; red and blue indicate positive and negative coefficients, respectively. Because observations from all four dietary treatments were pooled, the apparent correlations may primarily reflect separation among treatment groups along the dietary dose gradient rather than biological associations independent of treatment. The matrix is descriptive and does not establish causal relationships.

Figure 3 summarizes exploratory Spearman correlations among the nine pen-level variables. ADG was positively correlated with GSH-Px (*ρ* = 0.80) and negatively correlated with diarrhea rate (*ρ* = −0.62), UN (*ρ* = −0.74), TNF-α (*ρ* = −0.78), DAO (*ρ* = −0.77), and D-lactate (*ρ* = −0.71). GSH-Px was negatively correlated with diarrhea rate (*ρ* = −0.70), UN (*ρ* = −0.93), TNF-α (*ρ* = −0.96), DAO (*ρ* = −0.93), and D-lactate (*ρ* = −0.94). Because the 16 observations were pooled across the four dietary treatments, the apparent correlations may primarily reflect between-treatment separation along the dietary dose gradient rather than biological associations independent of treatment. Accordingly, the correlation matrix should be regarded as descriptive and should not be interpreted as evidence of causal relationships.

**Figure 4:**
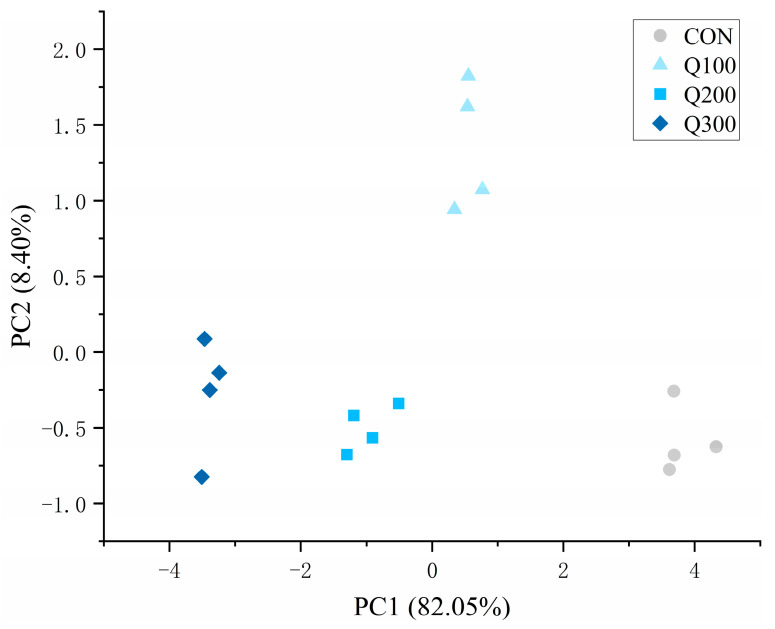
Exploratory PCA score plot based on z-score-standardized pen-level values for ADG, diarrhea rate, UN, MDA, GSH-Px, TNF-α, IL-10, DAO, and D-lactate. Each point represents one pen (four pens per treatment). PC1 and PC2 account for 82.05% and 8.40% of the variance, respectively. The plot is descriptive and does not demonstrate causal relationships.

Figure 4 shows the PCA of z-score-standardized pen-level observations. The first principal component (PC1) explained 82.05% of the total variance, and the second principal component (PC2) explained 8.40% (90.45% cumulative). Scores were separated predominantly along PC1: CON clustered at positive values, Q100 and Q200 occupied intermediate positions, and Q300 clustered at negative values. As with the correlation analysis, this visualization is descriptive and may partly reflect the dietary dose gradient rather than relationships independent of treatment.

**Figure 5:**
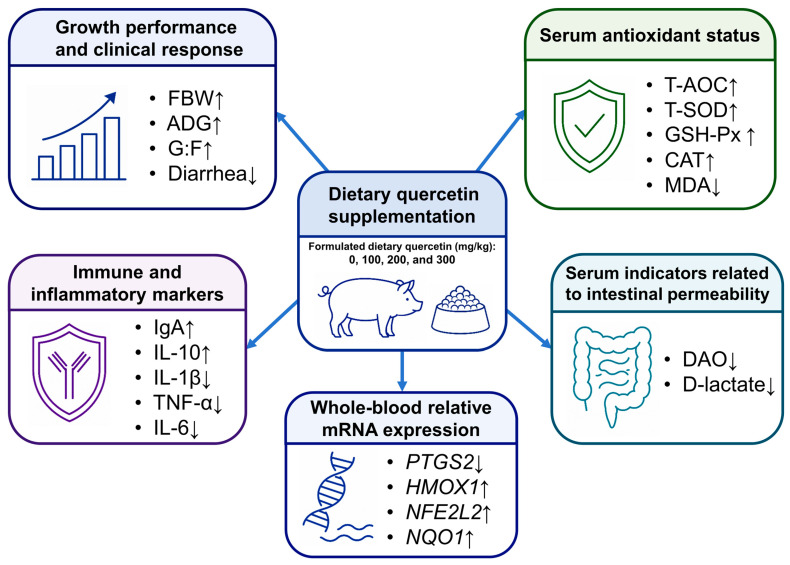
Schematic summary of the measured responses to graded dietary quercetin in weaned piglets. The scheme integrates growth, serum, and whole-blood measurements obtained after 28 days of feeding diets formulated to contain 0, 100, 200, or 300 mg/kg quercetin. Upward (↑) and downward (↓) arrows indicate significant positive and negative linear dose–response trends, respectively, across the graded quercetin inclusion levels. The connectors organize the measured domains and do not denote causal or tissue-specific mechanistic relationships.

An overview of the measured growth, serum, and whole-blood responses is provided in Figure 5. The connecting arrows organize the dietary treatment and measured response domains but do not imply causal mechanisms or verified tissue-specific signaling.

## 4. Discussion

### 4.1. Growth Performance, Diarrhea, and Serum Biochemical Responses

FBW was higher in Q200 and Q300 than in CON and Q100. ADG was higher in Q200 and Q300 than in CON and was also higher in Q300 than in Q100. G:F was higher in Q200 and Q300 than in CON and Q100 and was higher in Q300 than in Q200. Diarrhea rate was lower in Q300 than in CON and Q100 but did not differ significantly from Q200. However, the study was not designed to identify a universally optimal inclusion level.

Evidence from broader dose-ranging studies suggests that the response to dietary quercetin is not necessarily proportional to the inclusion level. Degroote et al. [6] evaluated dietary quercetin concentrations of 0, 100, 300, and 900 mg/kg and found that the 900 mg/kg group had greater day-42 body weight and overall average daily gain than the 300 mg/kg group; however, neither outcome differed significantly from the control group, and feed efficiency was unaffected. Thus, the highest tested dose did not produce a consistent overall growth advantage. By contrast, Mao et al. [7] evaluated graded concentrations up to 750 mg/kg and reported improved feed efficiency and reduced diarrhea incidence. These differing findings indicate that responses at higher inclusion levels may be endpoint-specific and influenced by the experimental context rather than increasing uniformly with dose. The present study focused on characterizing graded responses within a lower-to-moderate range of 0–300 mg/kg. Accordingly, the dose-responsive increases and decreases observed within this range should not be extrapolated beyond 300 mg/kg. Further studies incorporating broader dose gradients are needed to determine whether the responses persist, reach a plateau, or change at higher inclusion levels.

Other studies have also linked quercetin supplementation with immune or oxidative responses under challenge conditions [8,9]. Comparable changes in performance, redox status, and intestinal outcomes have been reported with plant-extract combinations, ferulic acid, apple polyphenols, silybin, and licorice flavonoids in nursery pigs [27,28,29,30,31]. Additional studies of tea-derived additives, algal polysaccharides, and probiotic–polysaccharide combinations have reported changes in growth, feed utilization, diarrhea, antioxidant, or immune outcomes [32,33,34,35,36]. Studies of berberine further illustrate the importance of dose and challenge context: protective intestinal responses were reported in enterotoxigenic *Escherichia coli* (ETEC)-challenged piglets, whereas a low dietary dose resulted in few detectable changes in gut morphology or microbiota in unchallenged piglets [37,38]. Differences in active compounds, basal diets, inclusion rates, animal age, and challenge status limit direct comparisons. The present findings therefore describe the responses to purified quercetin under the dietary and management conditions used here.

The serum biochemical changes accompanying the production responses were likewise treatment-specific. Compared with CON, ALB and TP were higher in Q100, Q200, and Q300, whereas UN, AST, TG, LDL-C, LDH, and GGT were lower in all three supplemented groups. ALT was lower in Q200 and Q300 than in CON and Q100 and was also lower in Q300 than in Q200. These shifts describe changes in circulating biochemical indices; however, serum enzyme activities and metabolite concentrations are physiological markers rather than direct measures of hepatic function, metabolic health, nutrient utilization, or disease resistance. The different dose–response shapes across serum endpoints may reflect differences in tissue origin, biological turnover, regulatory thresholds, and sensitivity to short-term physiological variation. ALP reached its lowest value in Q200 but returned toward the CON value in Q300, indicating both linear and quadratic components rather than a uniformly decreasing response. Because circulating ALP in pigs can vary with age and skeletal development, and because ALP isoenzymes were not measured in the present study, this pattern cannot be attributed specifically to hepatic effects [39,40]. CK also varied non-monotonically across treatments and showed neither a significant linear nor quadratic response. Serum CK is sensitive to muscular exertion and handling- or transport-related physiological stress in pigs [41]; therefore, the observed pattern may partly reflect transient muscular variation rather than a reproducible dose-dependent effect of quercetin, although this explanation was not directly tested in the present study. Consequently, the ALP and CK results should be regarded as endpoint-specific responses for which the underlying mechanisms remain unresolved.

By contrast, the clearer linear responses observed for TP, ALB, UN, AST, ALT, TG, LDL-C, LDH, and GGT indicate that these circulating measures varied more consistently across the formulated quercetin inclusion levels within the tested range. The concurrent increases in ALB and TP indicate changes in circulating protein measures, whereas the decrease in UN is compatible with altered amino acid utilization [42]. The lower AST, ALT, LDH, and GGT values indicate reduced circulating enzyme activities, but these serum measures do not identify the tissue source or establish improved hepatic function. The reductions in TG and LDL-C are compatible with altered lipid handling [28]. However, because nitrogen balance, nutrient digestibility, hepatic histology, and lipid-metabolism pathways were not measured, these interpretations remain provisional and do not establish improved nutrient utilization or changes in specific metabolic pathways. The contrast between monotonic and non-monotonic responses does not imply that the former are necessarily more biologically important; rather, individual biomarkers may respond at different thresholds and time scales. Given the single terminal sampling point and the limited number of independent pens, the present study cannot distinguish true biphasic responses from normal biological variation.

### 4.2. Antioxidant and Immune-Inflammatory Responses

Compared with CON, serum T-AOC, T-SOD, GSH-Px, and CAT were higher, whereas MDA was lower, in Q100, Q200, and Q300. Serum IL-1β, TNF-α, and IL-6 were lower, whereas IL-10 was higher, in each supplemented group than in CON. Serum IgA was higher in all three supplemented groups than in CON, whereas IgG and IgM were higher in Q100 and Q300 than in CON; Q200 did not differ from CON for either IgG or IgM. Together, these results describe coordinated but endpoint-specific variation in circulating redox, immunoglobulin, and inflammatory markers and are broadly consistent with observations from quercetin studies in weaned or challenged pigs [6,8,9]. Studies of Astragalus-ginseng polysaccharides, Isatidis root polysaccharides, plant polyphenol preparations, chlorogenic acid, and berberine have likewise reported changes in immune, oxidative, or intestinal endpoints in weaned pigs [43,44,45,46,47]. Nevertheless, concurrent shifts in these variables do not establish temporal order or direct causation.

Quercetin may influence redox status through modulation of endogenous antioxidant defenses. The concurrent increases in T-AOC, T-SOD, GRAC, GSH-Px, and CAT, together with the reduction in MDA, are compatible with enhanced antioxidant capacity and reduced lipid peroxidation. In porcine and IPEC-J2 models, quercetin has been associated with Nrf2-related cytoprotective responses [48,49]. The serum antioxidant profile and whole-blood transcript changes in the present study were directionally consistent with those reports; however, tissue-specific Nrf2 activation was not assessed, and the mechanism underlying the serum antioxidant responses therefore remains to be established.

The serum response was not identical across all endpoints or dose levels. POD decreased progressively with increasing quercetin inclusion, whereas Hs-CRP was highest in Q100 and returned to values comparable with CON in Q200 and Q300. This Hs-CRP pattern did not parallel the progressive reductions in TNF-α and IL-6. One possible explanation is that individual cytokines and acute-phase proteins reflect different components of the inflammatory response and may differ in their temporal profiles, production sites, and regulatory thresholds. An experimental study in pigs challenged with lipopolysaccharide demonstrated distinct temporal profiles for circulating cytokines and C-reactive protein, indicating that these markers need not vary synchronously [50].

The isolated increase in Hs-CRP at Q100, together with the significant quadratic contrast and the return to values comparable with CON at Q200 and Q300, indicates a non-monotonic response confined to the lowest supplemented dose. Because blood was collected only at the terminal sampling point, the present study cannot determine whether this pattern reflects normal biomarker variability, differences in response timing, or a reproducible low-dose effect. It should therefore not be interpreted as evidence that increasing quercetin inclusion promoted systemic inflammation. The lower POD activity should be interpreted alongside the concurrent reduction in MDA, the increase in T-AOC, and the higher activities of T-SOD, GSH-Px, and CAT. Overall, the serum response was coordinated across several related endpoints but was not uniform, emphasizing that different biomarkers may respond through distinct physiological processes and dose thresholds.

### 4.3. Serum Indicators Related to Intestinal Permeability

Serum DAO activity and D-lactate concentration are indirect circulating markers associated with intestinal permeability. Compared with CON, both variables were lower in Q100, Q200, and Q300 and decreased stepwise across the four dietary treatments. Accordingly, these results indicate lower circulating permeability-related markers following quercetin supplementation. Related intestinal barrier outcomes have been reported with a benzoic acid–*Bacillus coagulans*–oregano oil combination, ellagic acid, and the structurally related flavonol glycoside rutin in weaned piglets [51,52,53]. However, these circulating markers cannot localize the response to the intestine or establish its structural basis. Because intestinal morphology, villus architecture, tight-junction proteins, mucins, and histopathology were not assessed, and no direct in vivo permeability measurement was performed, the present findings do not demonstrate improved or restored intestinal barrier function.

### 4.4. Whole-Blood Gene Expression

Compared with CON, whole-blood *PTGS2* expression was lower in Q100, Q200, and Q300 and was lowest in Q300, whereas *SOD1* expression did not differ among treatments. *HMOX1* expression increased stepwise from CON through Q300. *NFE2L2* and *NQO1* expression were higher in all supplemented groups than in CON; *NFE2L2* was higher in Q200 and Q300 than in Q100, whereas *NQO1* was highest in Q300. These treatment-specific transcript patterns were broadly aligned with the serum redox and inflammatory findings. *PTGS2* encodes cyclooxygenase-2, an inducible enzyme involved in prostaglandin synthesis during inflammatory responses; thus, its lower whole-blood expression was directionally consistent with the reduced circulating concentrations of IL-1β, TNF-α, and IL-6. *NFE2L2* encodes Nrf2, whereas *HMOX1* and *NQO1* are commonly recognized as Nrf2-responsive cytoprotective genes. The concurrent increases in these transcripts were compatible with a coordinated whole-blood transcriptional response related to redox regulation. In contrast, the absence of a treatment effect on *SOD1* indicates that the transcriptional response was gene-specific rather than a uniform increase in all antioxidant-related genes. In challenged porcine models, quercetin attenuated deoxynivalenol-induced intestinal barrier dysfunction in the intestinal porcine epithelial cell line J2 (IPEC-J2) and in weaned piglets [48] and increased the mRNA expression of *NFE2L2*, *NQO1*, and superoxide dismutase 2 (*SOD2*), together with the protein abundance of Nrf2, HO-1, and NQO1, in IPEC-J2 cells exposed to zearalenone and lipopolysaccharide [49]. However, because only mRNA abundance was measured in circulating blood cells, these findings do not establish Nrf2 nuclear translocation, Nrf2-related protein abundance or activity, pathway activation in intestinal or hepatic tissues, or causal relationships between whole-blood transcripts and serum outcomes. They should therefore be interpreted as systemic transcriptional responses rather than tissue-specific mechanistic evidence.

### 4.5. Strengths and Limitations

Pen-level analysis was appropriate because the dietary treatments were assigned at the pen level. However, although 128 piglets were included, the effective number of independent experimental units was four pens per treatment. This relatively small number of independent replicates limits statistical power and the precision of the estimated treatment effects, particularly given the large number of biochemical, antioxidant, inflammatory, permeability-related, and molecular endpoints examined. Because numerous endpoints were analyzed, the possibility of false-positive findings across the full set of outcomes cannot be excluded. Tukey’s test controlled pairwise comparisons within each endpoint but did not address multiplicity across endpoints. Accordingly, the findings should be interpreted primarily on the basis of consistent response patterns across related variables rather than isolated statistically significant results.

The exploratory correlation and PCA analyses were based on only 16 pooled pen-level observations. These analyses should therefore be regarded as descriptive summaries. The apparent correlations and PCA score patterns may primarily reflect treatment-group differences along the dietary dose gradient rather than biological associations independent of treatment. Accordingly, these exploratory patterns do not establish causal relationships. Similarly, Figure 5 organizes the measured response domains only; its connectors do not imply mechanistic or causal relationships. Additional limitations include the absence of analytical confirmation of quercetin recovery, homogeneity, or stability in the finished diets, the lack of tissue-level intestinal measurements, and the use of whole blood rather than intestinal or hepatic tissue for transcript analysis. Although the commercial product was reported by the supplier to have an HPLC purity of at least 99.5%, quercetin was not independently quantified after diet mixing or storage. Subsequent studies should verify quercetin recovery, homogeneity, and stability in finished diets and incorporate tissue-level intestinal and molecular endpoints. They should also include a larger number of independent pens and use appropriately powered confirmatory designs [54]. Because the present experiment involved clinically healthy piglets without a disease challenge, extrapolation to high-pathogen-pressure conditions also requires caution.

## 5. Conclusions

In this 28-day feeding study, piglets fed diets formulated to contain 200 or 300 mg/kg quercetin had higher final body weight and average daily gain than CON piglets, and those fed the diet formulated to contain 300 mg/kg had the highest gain-to-feed ratio. Diarrhea rate decreased linearly with increasing quercetin inclusion, with Q300 showing a lower rate than CON and Q100. Quercetin supplementation was also associated with higher serum antioxidant capacity and activities of several antioxidant enzymes, lower malondialdehyde and pro-inflammatory cytokine concentrations, reduced serum DAO activity and D-lactate concentration, and coordinated changes in selected whole-blood transcripts. These findings support continued evaluation of quercetin as a feed additive for weaned piglets. Future studies should verify quercetin recovery, homogeneity, and stability in complete diets and investigate the tissue-level basis of the observed responses.

## Figures and Tables

**Table 1:** Ingredient composition and nutrient profile of the experimental diets (as-fed basis).

Item	CON	Q100	Q200	Q300
Ingredient composition, %				
Corn	11.04	11.04	11.04	11.04
Wheat middlings	1.96	1.96	1.96	1.96
Wheat flour	12.25	12.25	12.25	12.25
Rice bran meal	2.94	2.94	2.94	2.94
Extruded corn	26.95	26.95	26.95	26.95
Biscuit meal	14.70	14.70	14.70	14.70
Soybean meal	7.53	7.53	7.53	7.53
Extruded soybean	7.84	7.84	7.84	7.84
Fermented soybean meal	2.94	2.94	2.94	2.94
Fish meal	2.45	2.45	2.45	2.45
White sugar	1.96	1.96	1.96	1.96
Ground peanut hulls	2.03	2.02	2.01	2.00
Limestone	0.98	0.98	0.98	0.98
Dicalcium phosphate	0.29	0.29	0.29	0.29
Monocalcium phosphate	0.29	0.29	0.29	0.29
Sodium chloride	0.29	0.29	0.29	0.29
L-Lysine (98.5%)	0.60	0.60	0.60	0.60
DL-Methionine	0.10	0.10	0.10	0.10
L-Threonine	0.17	0.17	0.17	0.17
Citric acid monohydrate	0.98	0.98	0.98	0.98
Quercetin	0.00	0.01	0.02	0.03
Premix ^1^	1.71	1.71	1.71	1.71
Total	100.00	100.00	100.00	100.00
Nutrient composition ^2^				
Moisture, %	10.74	10.74	10.74	10.74
Digestible energy, MJ/kg	14.02	14.02	14.02	14.02
Crude protein, %	17.23	17.23	17.23	17.23
Ether extract, %	5.35	5.35	5.35	5.35
Crude fiber, %	2.86	2.86	2.86	2.86
Ash, %	4.59	4.59	4.59	4.59
Calcium, %	0.76	0.76	0.76	0.76
Total phosphorus, %	0.55	0.55	0.55	0.55
Available phosphorus, %	0.28	0.28	0.28	0.28
Total iron, mg/kg	150	150	150	150
Lysine, %	1.33	1.33	1.33	1.33
Methionine, %	0.39	0.39	0.39	0.39
Total sulfur amino acids, %	0.70	0.70	0.70	0.70
Threonine, %	0.80	0.80	0.80	0.80
Tryptophan, %	0.19	0.19	0.19	0.19

CON, diet without supplemental quercetin; Q100, Q200, and Q300, diets formulated to contain 100, 200, and 300 mg/kg quercetin, respectively. Quercetin replaced ground peanut hulls on an equal-mass basis. Quercetin inclusion rates of 0.01%, 0.02%, and 0.03% correspond to 100, 200, and 300 mg/kg diet, respectively. ^1^ Per kg of diet, the premix supplied vitamin A, 8000 IU; vitamin C, 500 mg; vitamin D3, 3000 IU; vitamin E, 60 mg; vitamin K, 35 mg; vitamin B1, 15 mg; vitamin B2, 30 mg; vitamin B6, 15 mg; vitamin B12, 0.5 mg; choline, 500 mg; nicotinamide, 175 mg; D-biotin, 2.5 mg; folic acid, 5 mg; pantothenic acid, 50 mg; Zn, 120 mg; Cu, 35 mg; Fe, 55 mg; Mn, 30 mg; Se, 0.3 mg; and I, 0.6 mg. ^2^ Crude protein, ether extract, and ash were determined analytically; the other nutrient values, including total dietary Fe, were calculated from ingredient composition. The premix supplied 55 mg/kg supplemental Fe, whereas the total dietary Fe concentration, including contributions from the basal ingredients, was calculated as 150 mg/kg in all treatments. Because quercetin replaced only 0.01–0.03% of the diet, the calculated nutrient values were identical among treatments at the reported precision.

**Table 2:** Growth performance, average daily feed intake, and diarrhea rate of weaned piglets receiving graded dietary quercetin.

Item	CON	Q100	Q200	Q300	*p*-Treatment	*η_p_* *^2^*	*p*-Linear	*p*-Quadratic
IBW (kg)	10.28 ± 0.08	10.23 ± 0.06	10.28 ± 0.10	10.34 ± 0.10	0.867	0.056	0.582	0.562
FBW (kg)	18.53 ± 0.08 ^b^	18.60 ± 0.12 ^b^	18.97 ± 0.08 ^a^	19.15 ± 0.07 ^a^	<0.001	0.741	<0.001	0.552
ADG (g/d)	294.6 ± 4.0 ^c^	298.8 ± 3.1 ^bc^	310.5 ± 3.3 ^ab^	314.8 ± 2.1 ^a^	0.002	0.686	<0.001	0.979
ADFI (g/d)	588.0 ± 5.9 ^a^	560.0 ± 6.5 ^a^	514.4 ± 8.2 ^b^	472.3 ± 9.5 ^c^	<0.001	0.917	<0.001	0.375
G:F	0.50 ± 0.01 ^c^	0.53 ± 0.01 ^c^	0.60 ± 0.01 ^b^	0.67 ± 0.01 ^a^	<0.001	0.920	<0.001	0.184
Diarrhea rate (%)	21.8 ± 1.6 ^a^	19.3 ± 1.3 ^a^	16.9 ± 1.2 ^ab^	13.5 ± 1.1 ^b^	0.004	0.652	<0.001	0.633

CON, basal diet without added quercetin; Q100, Q200, and Q300, basal diets formulated to contain 100, 200, and 300 mg/kg quercetin, respectively. Values are means ± SEM (*n* = 4 pens per treatment). For variables with a significant overall treatment effect, means within a row that do not share a common superscript letters (a–c) differ according to Tukey’s multiple-comparison test (*p* < 0.05). Diarrhea-rate data were subjected to an arcsine square-root transformation before statistical analysis; untransformed percentages are presented for ease of interpretation. *p*-treatment was obtained from one-way ANOVA, and *p*-linear and *p*-quadratic were obtained from orthogonal polynomial contrasts. *η_p_^2^* denotes partial eta squared for the overall dietary treatment effect. For diarrhea rate, *η_p_^2^* was calculated using the arcsine square-root-transformed proportions. IBW, initial body weight; FBW, final body weight; ADG, average daily gain; ADFI, average daily feed intake; G:F, gain-to-feed ratio.

**Table 3:** Serum biochemical indices in weaned piglets fed diets with graded quercetin concentrations.

Item	CON	Q100	Q200	Q300	*p*-Treatment	*p*-Linear	*p*-Quadratic
ALB (g/L)	25.28 ± 0.07 ^d^	25.96 ± 0.08 ^c^	27.02 ± 0.05 ^b^	28.05 ± 0.09 ^a^	<0.001	<0.001	0.042
TP (g/L)	53.6 ± 0.5 ^c^	58.9 ± 0.4 ^b^	61.8 ± 0.5 ^a^	62.7 ± 0.4 ^a^	<0.001	<0.001	<0.001
GLU (mmol/L)	6.67 ± 0.05 ^b^	6.91 ± 0.03 ^ab^	7.11 ± 0.11 ^a^	7.14 ± 0.08 ^a^	0.003	<0.001	0.226
UN (mmol/L)	2.67 ± 0.01 ^a^	2.38 ± 0.01 ^b^	2.28 ± 0.03 ^c^	2.18 ± 0.02 ^d^	<0.001	<0.001	<0.001
TBIL (µmol/L)	12.27 ± 0.22	12.03 ± 0.10	12.19 ± 0.14	12.21 ± 0.15	0.754	0.983	0.437
DBIL (µmol/L)	4.90 ± 0.03	4.88 ± 0.06	4.74 ± 0.05	4.69 ± 0.10	0.131	0.027	0.871
ALP (King units/100 mL)	13.62 ± 0.18 ^a^	13.18 ± 0.06 ^a^	12.31 ± 0.11 ^b^	13.32 ± 0.12 ^a^	<0.001	0.009	<0.001
AST (U/L)	78.8 ± 0.4 ^a^	72.0 ± 0.4 ^b^	70.1 ± 0.5 ^c^	67.2 ± 0.3 ^d^	<0.001	<0.001	<0.001
ALT (U/L)	47.2 ± 0.4 ^a^	46.4 ± 0.3 ^a^	43.7 ± 0.3 ^b^	41.8 ± 0.2 ^c^	<0.001	<0.001	0.113
CK (U/mL)	1.67 ± 0.03 ^b^	1.79 ± 0.01 ^a^	1.57 ± 0.02 ^c^	1.74 ± 0.01 ^ab^	<0.001	0.968	0.229
CREA (µmol/L)	98.1 ± 0.9 ^a^	95.5 ± 0.9 ^a^	91.2 ± 1.1 ^b^	86.6 ± 0.9 ^c^	<0.001	<0.001	0.321
TG (mmol/L)	0.80 ± 0.01 ^a^	0.72 ± 0.01 ^b^	0.62 ± 0.02 ^c^	0.52 ± 0.01 ^d^	<0.001	<0.001	0.358
TC (mmol/L)	2.32 ± 0.02 ^a^	2.18 ± 0.02 ^b^	2.08 ± 0.03 ^b^	1.84 ± 0.02 ^c^	<0.001	<0.001	0.050
HDL-C (mmol/L)	0.73 ± 0.01 ^d^	0.78 ± 0.01 ^c^	0.86 ± 0.01 ^b^	0.94 ± 0.01 ^a^	<0.001	<0.001	0.282
LDL-C (mmol/L)	0.95 ± 0.01 ^a^	0.89 ± 0.01 ^b^	0.84 ± 0.01 ^c^	0.74 ± 0.01 ^d^	<0.001	<0.001	0.102
LDH (U/L)	871.0 ± 7.5 ^a^	827.9 ± 7.8 ^b^	770.0 ± 9.7 ^c^	694.3 ± 5.9 ^d^	<0.001	<0.001	0.060
GGT (U/L)	28.1 ± 0.5 ^a^	26.1 ± 0.5 ^b^	23.7 ± 0.4 ^c^	20.4 ± 0.5 ^d^	<0.001	<0.001	0.183

Values are means ± SEM (*n* = 4 pens per treatment). For variables with a significant overall treatment effect, means within a row that do not share a common superscript letters (a–d) differ according to Tukey’s multiple-comparison test (*p* < 0.05). ALB, albumin; TP, total protein; GLU, glucose; UN, urea nitrogen; TBIL, total bilirubin; DBIL, direct bilirubin; ALP, alkaline phosphatase; AST, aspartate aminotransferase; ALT, alanine aminotransferase; CK, creatine kinase; CREA, creatinine; TG, triglyceride; TC, total cholesterol; HDL-C, high-density lipoprotein cholesterol; LDL-C, low-density lipoprotein cholesterol; LDH, lactate dehydrogenase; GGT, γ-glutamyl transferase. ALP activity is expressed as King units/100 mL, in accordance with the assay kit instructions. *p*-treatment was obtained from one-way ANOVA; *p*-linear and *p*-quadratic were obtained from orthogonal polynomial contrasts.

**Table 4:** Serum antioxidant indices of weaned piglets receiving graded dietary quercetin.

Item	CON	Q100	Q200	Q300	*p*-Treatment	*p*-Linear	*p*-Quadratic
T-AOC (U/mL)	0.64 ± 0.01 ^c^	0.76 ± 0.01 ^b^	0.75 ± 0.01 ^b^	0.79 ± 0.01 ^a^	<0.001	<0.001	<0.001
T-SOD (U/mL)	16.4 ± 0.2 ^b^	18.8 ± 0.2 ^a^	18.6 ± 0.2 ^a^	19.3 ± 0.2 ^a^	<0.001	<0.001	<0.001
POD (U/mL)	173.3 ± 1.4 ^a^	159.0 ± 1.5 ^b^	154.8 ± 2.0 ^b^	145.9 ± 1.5 ^c^	<0.001	<0.001	0.113
MDA (nmol/mL)	3.20 ± 0.02 ^a^	2.60 ± 0.02 ^b^	2.70 ± 0.03 ^b^	2.48 ± 0.03 ^c^	<0.001	<0.001	<0.001
GRAC (unitless)	9.32 ± 0.09 ^c^	9.91 ± 0.07 ^b^	10.11 ± 0.07 ^b^	10.64 ± 0.06 ^a^	<0.001	<0.001	0.723
GSH-Px (U/mL)	468.0 ± 7.6 ^d^	534.0 ± 6.3 ^c^	639.1 ± 13.3 ^b^	786.3 ± 8.9 ^a^	<0.001	<0.001	<0.001
CAT (U/mL)	12.7 ± 0.2 ^d^	14.6 ± 0.2 ^c^	16.6 ± 0.2 ^b^	19.5 ± 0.4 ^a^	<0.001	<0.001	0.065

Values are means ± SEM (*n* = 4 pens per treatment). For variables with a significant overall treatment effect, means within a row that do not share a common superscript letters (a–d) differ according to Tukey’s multiple-comparison test (*p* < 0.05). T-AOC, total antioxidant capacity; T-SOD, total superoxide dismutase; POD, peroxidase; MDA, malondialdehyde; GRAC, glutathione reductase activity coefficient; GSH-Px, glutathione peroxidase; CAT, catalase. *p*-treatment was obtained from one-way ANOVA, and *p*-linear and *p*-quadratic were obtained from orthogonal polynomial contrasts. For contextual comparison, a previous study of unchallenged weaned pigs reported control-group serum values of 2.10 ± 0.10 U/mL for T-AOC, 18.15 ± 0.37 U/mL for T-SOD, 3.68 ± 0.40 nmol/mL for MDA, and 18.89 ± 1.26 U/mL for CAT [25]. These study-specific values are provided only as contextual benchmarks and should not be interpreted as universal physiological reference intervals.

**Table 5:** Immune and inflammatory serum variables in weaned piglets fed graded dietary quercetin.

Item	CON	Q100	Q200	Q300	*p*-Treatment	*p*-Linear	*p*-Quadratic
IgA (µg/mL)	44.3 ± 0.6 ^b^	46.6 ± 0.3 ^a^	46.5 ± 0.3 ^a^	47.6 ± 0.3 ^a^	<0.001	<0.001	0.142
IgG (µg/mL)	503.38 ± 3.34 ^b^	514.94 ± 0.85 ^a^	510.39 ± 0.07 ^ab^	517.48 ± 0.04 ^a^	<0.001	<0.001	0.218
IgM (µg/mL)	55.8 ± 0.5 ^b^	57.6 ± 0.2 ^a^	56.5 ± 0.4 ^ab^	57.9 ± 0.4 ^a^	0.006	0.011	0.607
IL-1β (ng/L)	58.1 ± 0.4 ^a^	52.5 ± 0.5 ^b^	52.1 ± 0.2 ^b^	52.8 ± 0.3 ^b^	<0.001	<0.001	<0.001
IL-10 (ng/L)	209.6 ± 1.1 ^c^	221.8 ± 0.8 ^a^	216.4 ± 0.6 ^b^	222.6 ± 0.7 ^a^	<0.001	<0.001	0.004
Hs-CRP (mg/L)	2.82 ± 0.02 ^b^	3.24 ± 0.10 ^a^	2.80 ± 0.02 ^b^	2.76 ± 0.02 ^b^	<0.001	0.020	<0.001
LZM (µg/L)	97.3 ± 0.8	98.1 ± 0.5	97.7 ± 0.4	98.6 ± 0.3	0.389	0.165	0.971
TNF-α (pg/mL)	125.9 ± 3.4 ^a^	100.5 ± 2.1 ^b^	81.3 ± 1.3 ^c^	62.2 ± 1.7 ^d^	<0.001	<0.001	0.191
IL-6 (pg/mL)	74.9 ± 2.4 ^a^	59.7 ± 0.8 ^b^	50.0 ± 0.7 ^c^	39.6 ± 0.7 ^d^	<0.001	<0.001	0.101

Values are means ± SEM (*n* = 4 pens per treatment). For variables with a significant overall treatment effect, means within a row that do not share a common superscript letters (a–d) differ according to Tukey’s multiple-comparison test (*p* < 0.05). IgA, immunoglobulin A; IgG, immunoglobulin G; IgM, immunoglobulin M; IL-1β, interleukin-1β; IL-10, interleukin-10; Hs-CRP, high-sensitivity C-reactive protein; LZM, lysozyme; TNF-α, tumor necrosis factor-α; IL-6, interleukin-6. *p*-treatment was obtained from one-way ANOVA, and *p*-linear and *p*-quadratic were obtained from orthogonal polynomial contrasts. For contextual comparison, published age-specific 95% reference intervals for serum TNF-α in healthy piglets were 22.0–108.4, 51.9–162.4, and 46.3–202.6 pg/mL at 21, 28, and 35 days of age, respectively [26]. Because these intervals vary with age, population, and analytical method, they are provided only for contextual interpretation and are not directly applicable to the present study.

## Data Availability

Pen-level data underlying the results are provided in the Supplementary Materials. Additional study information may be requested from the corresponding authors.

## Supplementary Materials

The following supporting information can be downloaded at: https://www.mdpi.com/article/10.3390/ani16152429/s1, Table S1: Primer sequences, amplicon information, amplification efficiencies, and coefficients of determination used for RT-qPCR analysis; Table S2: Reverse-transcription and real-time PCR reaction conditions; Table S3A: Pen-level dataset: growth performance, average daily feed intake, and diarrhea rate; Table S3B: Pen-level dataset: serum biochemical variables; Table S3C: Pen-level dataset: serum antioxidant and immune/inflammatory variables; Table S3D: Pen-level dataset: serum indicators related to intestinal permeability; Table S3E: Pen-level dataset: whole-blood relative mRNA expression; Table S3F: Linear and quadratic contrast results for all endpoints; Table S4: Analytical endpoints, measurement methods, and reported units.

## Abbreviations

*ACTB*, actin beta; ADFI, average daily feed intake; ADG, average daily gain; ALB, albumin; ALP, alkaline phosphatase; ALT, alanine aminotransferase; ANOVA, analysis of variance; AST, aspartate aminotransferase; CAT, catalase; cDNA, complementary DNA; CK, creatine kinase; CON, basal diet without supplemental quercetin; COX-2, cyclooxygenase-2; CREA, creatinine; DAO, diamine oxidase; DBIL, direct bilirubin; ETEC, enterotoxigenic *Escherichia coli*; FBW, final body weight; G:F, gain-to-feed ratio; GGT, γ-glutamyl transferase; GLU, glucose; GRAC, glutathione reductase activity coefficient; GSH-Px, glutathione peroxidase; HDL-C, high-density lipoprotein cholesterol; *HMOX1*, heme oxygenase 1; HO-1, heme oxygenase-1; HPLC, high-performance liquid chromatography; Hs-CRP, high-sensitivity C-reactive protein; IBW, initial body weight; IgA, immunoglobulin A; IgG, immunoglobulin G; IgM, immunoglobulin M; IL-1β, interleukin-1β; IL-6, interleukin-6; IL-10, interleukin-10; IPEC-J2, intestinal porcine epithelial cell line J2; K_2_EDTA, dipotassium ethylenediaminetetraacetate; LDH, lactate dehydrogenase; LDL-C, low-density lipoprotein cholesterol; LZM, lysozyme; MDA, malondialdehyde; mRNA, messenger RNA; *NFE2L2*, nuclear factor erythroid 2-related factor 2; *NQO1*, NAD(P)H quinone dehydrogenase 1; NRC, National Research Council; Nrf2, nuclear factor erythroid 2-related factor 2; PC1, first principal component; PC2, second principal component; PCA, principal component analysis; PCR, polymerase chain reaction; POD, peroxidase; *PTGS2*, prostaglandin-endoperoxide synthase 2; qPCR, quantitative polymerase chain reaction; Q100, diet formulated to contain 100 mg/kg quercetin; Q200, diet formulated to contain 200 mg/kg quercetin; Q300, diet formulated to contain 300 mg/kg quercetin; RNA, ribonucleic acid; RT-qPCR, reverse-transcription quantitative polymerase chain reaction; SEM, standard error of the mean; *SOD1*, superoxide dismutase 1; *SOD2*, superoxide dismutase 2; T-AOC, total antioxidant capacity; TBIL, total bilirubin; TC, total cholesterol; TG, triglyceride; TNF-α, tumor necrosis factor-α; TP, total protein; T-SOD, total superoxide dismutase; UN, urea nitrogen.
