# Supplementary material for: Effects of Dietary Quercetin Supplementation on Growth Performance, Serum Health Indices, and Whole-Blood Gene Expression in Weaned Piglets
Source: Animals (Basel). 2026 Aug 6;16(15):2429. doi: 10.3390/ani16152429 (PMC13465313; doi:10.3390/ani16152429)
Supplement: Supplementary file 1 [file animals-16-02429-s001.zip › animals-4437639-supplementary.pdf]

Table S1. Primer sequences, amplicon information, amplification efficiencies, and coefficients of determination used for RT-qPCR analysis.

Gene symbol (common name)	Forward primer (5′–3′)	Reverse primer (5′–3′)	Amplicon size, bp	Annealing temperature, °C	Amplification efficiency (%)	R^2
ACTB (β-actin)	CGTCCACCGCAAATGCTTC	AACCGACTGCTGTCACCTTCAC	217	60	98.6	0.998
PTGS2 (COX-2)	CTTCCTCCTGTGCCTGATGACTG	GGTCTCTGCTTCTGATCTGTCTTG	200	60	96.2	0.996
SOD1	AGCCTGCTGGTTGTAGACAT	TCACCACAGGTACTGAAAGCA	102	60	98.1	0.997
HMOX1 (HO-1)	TCCTGGCTCAGCCTCAAATG	CGTTAAACACCTCCCTCCCC	108	60	95.7	0.995
NFE2L2 (Nrf2)	GCCCCTGGAAGCGTTAAAC	GGACTGTATCCCCAGAAGGTTGT	67	60	97.3	0.996
NQO1	GGTTTGGAGTCCCTGCCATT	ACCAGTGGTGATGGAAAGCA	134	60	96.9	0.997

ACTB, actin beta; *PTGS2*, prostaglandin-endoperoxide synthase 2; *SOD1*, superoxide dismutase 1; *HMOX1*, heme oxygenase 1; *NFE2L2*, nuclear factor erythroid 2-related factor 2; *NQO1*, NAD(P)H quinone dehydrogenase 1. Amplification efficiency and R^2 values were determined by standard-curve analysis.

Table S2. Reverse-transcription and real-time PCR reaction conditions.

Stage	Component or step	Amount / condition	Final concentration / comment
Reverse transcription	Total RNA	500 ng	Template RNA input
Reverse transcription	5× PrimeScript RT Master Mix (Perfect Real Time)	2.0 μL	1×
Reverse transcription	RNase-free water	Up to 10 μL	Final reaction volume: 10 μL
Reverse transcription	Reverse transcription program	37 °C for 15 min; 85 °C for 5 s; hold at 4 °C	
qPCR reaction	TB Green Premix Ex Taq II (Tli RNase H Plus; 2×)	10.0 μL	1×
qPCR reaction	Forward primer (10 μM)	0.8 μL	0.4 μM
qPCR reaction	Reverse primer (10 μM)	0.8 μL	0.4 μM
qPCR reaction	ROX Reference Dye II (50×)	0.4 μL	1×
qPCR reaction	cDNA template	1.0 μL	
qPCR reaction	RNase-free water	7.0 μL	Final reaction volume: 20 μL
Thermal cycling	Initial denaturation	95 °C for 30 s	
Thermal cycling	Amplification	39 cycles: 95 °C for 5 s and 60 °C for 30 s	Fluorescence acquired at annealing/extension
Melt curve	Dissociation analysis	60–95 °C, 0.5 °C increments, 5 s per increment	
Data acquisition	Instrument	Accurate 96 real-time PCR system	
Data acquisition	Technical replication	Triplicate reactions per sample	
Data analysis	Normalization and quantification	*ACTB* as the reference gene; mean ΔCt value of the CON group as the calibrator; $2^{-\Delta\Delta Ct}$ method.	

Table S3A. Pen-level dataset: growth performance, average daily feed intake, and diarrhea rate.

Group	Pen	IBW, kg	FBW, kg	ADG, g/d	ADFI, g/d	G:F	Diarrhea rate, %
CON	1	10.08	18.37	296.07	586.22	0.505	18.30
CON	2	10.35	18.57	293.57	595.95	0.493	22.32
CON	3	10.22	18.74	304.29	572.07	0.532	25.89
CON	4	10.46	18.43	284.64	597.74	0.476	20.54
Q100	1	10.12	18.64	304.29	562.94	0.541	16.52
Q100	2	10.28	18.55	295.36	567.09	0.521	19.20
Q100	3	10.38	18.89	303.93	541.00	0.562	22.77
Q100	4	10.15	18.32	291.79	568.99	0.513	18.75
Q200	1	10.18	19.04	316.43	522.11	0.606	14.29
Q200	2	10.33	19.12	313.93	496.01	0.633	17.86
Q200	3	10.08	18.76	310.00	533.20	0.581	19.64
Q200	4	10.52	18.96	301.43	506.40	0.595	15.63
Q300	1	10.24	19.20	320.00	486.40	0.658	11.16
Q300	2	10.40	19.18	313.57	464.08	0.676	13.84
Q300	3	10.12	18.96	315.71	489.35	0.645	16.52
Q300	4	10.58	19.26	310.00	449.50	0.690	12.50

The pen was the experimental unit for growth performance, feed intake, and diarrhea rate (n = 4 pens per treatment). Diarrhea rate (%) was calculated as (diarrheal pig-days / total pig-days) × 100; each pen represented 224 pig-days (8 piglets × 28 days).

Table S3B. Pen-level dataset: serum biochemical variables (Part 1 of 2).

Group	Pen	ALB (g/L)	TP (g/L)	GLU (mmol/L)	UN (mmol/L)	TBIL (μmol/L)	DBIL (μmol/L)	ALP (King units/ 100 mL)	AST (U/L)	ALT (U/L)
CON	1	25.50	52.73	6.565	2.710	12.89	4.850	14.06	78.90	47.90
CON	2	25.21	52.56	6.615	2.655	12.06	4.840	13.17	79.50	47.95
CON	3	25.23	54.40	6.755	2.670	12.22	4.965	13.56	77.78	46.23
CON	4	25.17	54.56	6.760	2.650	11.89	4.945	13.67	79.00	46.90
Q100	1	26.11	58.23	6.895	2.415	11.89	4.950	13.22	71.95	46.51
Q100	2	25.95	58.56	6.885	2.350	12.34	5.005	13.06	72.12	46.45
Q100	3	25.73	59.90	6.855	2.355	11.89	4.840	13.34	71.06	47.17
Q100	4	26.05	58.83	6.995	2.380	12.00	4.720	13.10	72.88	45.62
Q200	1	26.95	60.73	7.075	2.310	11.84	4.730	12.62	70.73	43.90
Q200	2	26.95	63.12	6.965	2.200	12.28	4.725	12.22	69.72	44.23
Q200	3	27.00	62.01	6.945	2.270	12.12	4.620	12.18	68.84	43.95
Q200	4	27.16	61.17	7.435	2.345	12.51	4.875	12.20	71.25	42.69
Q300	1	27.84	62.06	7.230	2.215	12.12	4.840	13.51	68.00	41.95
Q300	2	28.00	63.78	7.130	2.115	12.01	4.850	13.29	66.50	41.84
Q300	3	28.06	62.67	6.915	2.170	12.06	4.675	13.51	66.90	42.01
Q300	4	28.27	62.44	7.300	2.220	12.64	4.410	12.99	67.41	41.35

For serum endpoints, values from two sampled piglets within each pen were averaged before statistical analysis. Tables S3B (Parts 1 and 2) together constitute the complete dataset for serum biochemical variables.

Table S3B. Pen-level dataset: serum biochemical variables (Part 2 of 2).

Group	Pen	CK (U/mL)	CREA (μmol/L)	TG (mmol/L)	TC (mmol/L)	HDL-C (mmol/L)	LDL-C (mmol/L)	LDH (U/L)	GGT (U/L)
CON	1	1.670	100.39	0.795	2.315	0.735	0.950	866.39	29.34
CON	2	1.700	97.56	0.820	2.350	0.715	0.970	881.00	28.61
CON	3	1.595	95.95	0.775	2.270	0.745	0.930	851.95	27.11
CON	4	1.715	98.50	0.795	2.340	0.725	0.960	884.62	27.50
Q100	1	1.785	96.50	0.730	2.200	0.770	0.900	839.12	26.73
Q100	2	1.795	93.62	0.710	2.160	0.795	0.880	816.39	25.73
Q100	3	1.765	97.45	0.745	2.220	0.750	0.920	843.50	27.11
Q100	4	1.820	94.28	0.695	2.130	0.815	0.865	812.67	24.95
Q200	1	1.500	93.45	0.650	2.140	0.830	0.870	786.39	24.45
Q200	2	1.580	90.62	0.610	2.060	0.870	0.830	761.67	23.50
Q200	3	1.590	92.17	0.645	2.120	0.845	0.860	785.50	24.17
Q200	4	1.610	88.56	0.570	2.000	0.890	0.810	746.39	22.68
Q300	1	1.750	85.67	0.510	1.825	0.950	0.740	693.45	19.95
Q300	2	1.760	87.23	0.525	1.860	0.930	0.750	699.06	20.95
Q300	3	1.700	84.62	0.490	1.780	0.960	0.720	678.45	19.17
Q300	4	1.760	88.84	0.545	1.880	0.910	0.760	706.29	21.34

For serum endpoints, values from two sampled piglets within each pen were averaged before statistical analysis. Tables S3B (Parts 1 and 2) together constitute the complete dataset for serum biochemical variables.

Table S3C. Pen-level dataset: serum antioxidant and immune/inflammatory variables (Part 1 of 2).

Group	Pen	T-AOC (U/mL)	T-SOD (U/mL)	POD (U/mL)	MDA (nmol/mL)	GRAC (unitless)	GSH-Px (U/mL)	CAT (U/mL)
CON	1	0.650	15.95	171.95	3.17	9.05	460.67	13.05
CON	2	0.630	16.74	175.95	3.22	9.34	477.23	12.84
CON	3	0.640	16.25	170.17	3.16	9.45	483.67	12.12
CON	4	0.630	16.79	175.28	3.27	9.44	450.50	12.84
Q100	1	0.740	18.79	160.50	2.65	9.83	531.39	14.89
Q100	2	0.765	19.11	155.62	2.56	10.05	532.84	14.34
Q100	3	0.750	18.32	162.34	2.61	9.77	551.00	14.79
Q100	4	0.765	19.13	157.50	2.58	9.99	520.62	14.29
Q200	1	0.765	18.89	151.40	2.65	10.20	636.89	17.06
Q200	2	0.745	18.32	156.45	2.73	10.02	658.56	16.23
Q200	3	0.755	18.89	151.56	2.66	10.27	658.56	16.62
Q200	4	0.735	18.23	159.62	2.77	9.95	602.45	16.56
Q300	1	0.785	19.23	149.00	2.52	10.62	777.00	19.11
Q300	2	0.795	19.45	144.34	2.45	10.51	797.34	19.61
Q300	3	0.775	18.89	147.50	2.55	10.66	766.12	20.57
Q300	4	0.805	19.79	142.67	2.42	10.78	804.62	18.73

For serum endpoints, values from two sampled piglets within each pen were averaged before statistical analysis. Tables S3C (Parts 1 and 2) together constitute the complete dataset for serum antioxidant and immune/inflammatory variables.

Table S3C. Pen-level dataset: serum antioxidant and immune/inflammatory variables (Part 2 of 2).

Group	Pen	IgA (μg/mL)	IgG (μg/mL)	IgM (μg/mL)	IL-1β (ng/L)	IL-10 (ng/L)	Hs-CRP (mg/L)	LZM (μg/L)	TNF-α (pg/mL)	IL-6 (pg/mL)
CON	1	42.84	505.56	54.78	58.73	209.40	2.825	96.45	125.50	68.90
CON	2	44.84	493.45	55.95	58.23	209.17	2.765	95.40	116.61	80.50
CON	3	45.45	507.56	56.95	57.12	212.50	2.855	98.50	129.22	73.90
CON	4	44.17	506.95	55.62	58.51	207.22	2.820	98.67	132.06	76.40
Q100	1	46.40	515.95	57.45	52.62	221.95	3.005	99.45	101.95	60.56
Q100	2	47.12	512.45	58.23	51.23	221.95	3.330	97.95	95.62	57.90
Q100	3	45.95	515.28	57.45	53.78	219.56	3.450	98.22	105.45	61.23
Q100	4	46.73	516.06	57.28	52.28	223.56	3.185	96.95	99.00	59.01
Q200	1	47.12	510.50	57.45	52.40	217.00	2.835	97.17	81.90	50.51
Q200	2	46.95	510.50	56.78	51.62	217.34	2.730	97.23	77.62	47.84
Q200	3	46.12	510.34	55.73	52.51	214.62	2.830	97.56	82.90	51.01
Q200	4	45.95	510.23	55.90	51.67	216.50	2.815	99.00	82.95	50.62
Q300	1	47.56	517.45	57.90	53.23	221.06	2.765	99.34	59.01	40.45
Q300	2	48.34	517.50	58.67	52.62	223.90	2.710	98.50	63.62	38.01
Q300	3	47.67	517.39	57.95	51.95	223.62	2.810	97.84	66.23	41.17
Q300	4	46.73	517.56	56.90	53.23	221.72	2.745	98.67	59.95	38.84

For serum endpoints, values from two sampled piglets within each pen were averaged before statistical analysis. Tables S3C (Parts 1 and 2) together constitute the complete dataset for serum antioxidant and immune/inflammatory variables. T-AOC, total antioxidant capacity; T-SOD, total superoxide dismutase; POD, peroxidase; MDA, malondialdehyde; GRAC, glutathione reductase activity coefficient; GSH-Px, glutathione peroxidase; CAT, catalase; Hs-CRP, high-sensitivity C-reactive protein; LZM, lysozyme.

Table S3D. Pen-level dataset: serum indicators related to intestinal permeability.

Group	Pen	DAO (U/mL)	D-lactate (mmol/L)
CON	1	7.725	2.260
CON	2	7.120	2.385
CON	3	7.785	2.235
CON	4	7.505	2.300
Q100	1	6.615	2.050
Q100	2	7.005	1.965
Q100	3	6.505	2.065
Q100	4	6.785	1.895
Q200	1	5.725	1.715
Q200	2	5.950	1.670
Q200	3	5.505	1.565
Q200	4	5.730	1.635
Q300	1	4.725	1.415
Q300	2	4.950	1.400
Q300	3	4.505	1.265
Q300	4	4.730	1.350

For serum endpoints, values from two sampled piglets within each pen were averaged before statistical analysis.

Table S3E. Pen-level dataset: whole-blood relative mRNA expression.

**Group**	**Pen**	*PTGS2* (COX-2)	*SOD1*	*HMOX1* (HO-1)	*NFE2L2* (Nrf2)	*NQO1*
CON	1	1.000	1.030	1.030	0.920	0.930
CON	2	1.005	0.960	1.020	1.020	0.940
CON	3	1.080	1.020	1.020	1.000	0.930
CON	4	0.935	1.000	0.980	0.880	0.840
Q100	1	0.830	0.970	1.260	1.200	1.335
Q100	2	0.890	1.020	1.370	1.230	1.220
Q100	3	0.840	0.950	1.250	1.270	1.280
Q100	4	0.930	1.050	1.310	1.320	1.280
Q200	1	0.880	1.020	1.470	1.510	1.300
Q200	2	0.900	0.980	1.530	1.520	1.330
Q200	3	0.800	1.030	1.540	1.410	1.350
Q200	4	0.910	0.980	1.460	1.570	1.250
Q300	1	0.700	0.980	1.620	1.500	1.660
Q300	2	0.720	0.980	1.700	1.530	1.750
Q300	3	0.630	0.920	1.770	1.550	1.710
Q300	4	0.720	1.020	1.660	1.620	1.850

Each row represents one pen. For whole-blood endpoints, values from the two sampled piglets within each pen were averaged before analysis.

Table S3F. Linear and quadratic contrast results for all endpoints.

Outcome category	Variable	*p*-treatment	*p*-linear	*p*-quadratic
Growth performance	IBW (kg)	0.867	0.582	0.562
Growth performance	FBW (kg)	<0.001	<0.001	0.552
Growth performance	ADG (g/d)	0.002	<0.001	0.979
Growth performance	ADFI (g/d)	<0.001	<0.001	0.375
Growth performance	G:F	<0.001	<0.001	0.184
Growth performance	Diarrhea rate (%)	0.004	<0.001	0.633
Serum biochemistry	ALB (g/L)	<0.001	<0.001	0.042
Serum biochemistry	TP (g/L)	<0.001	<0.001	<0.001
Serum biochemistry	GLU (mmol/L)	0.003	<0.001	0.226
Serum biochemistry	UN (mmol/L)	<0.001	<0.001	<0.001
Serum biochemistry	TBIL (μmol/L)	0.754	0.983	0.437
Serum biochemistry	DBIL (μmol/L)	0.131	0.027	0.871
Serum biochemistry	ALP (King units/100 mL)	<0.001	0.009	<0.001
Serum biochemistry	AST (U/L)	<0.001	<0.001	<0.001
Serum biochemistry	ALT (U/L)	<0.001	<0.001	0.113
Serum biochemistry	CK (U/mL)	<0.001	0.968	0.229
Serum biochemistry	CREA (μmol/L)	<0.001	<0.001	0.321
Serum biochemistry	TG (mmol/L)	<0.001	<0.001	0.358
Serum biochemistry	TC (mmol/L)	<0.001	<0.001	0.050
Serum biochemistry	HDL-C (mmol/L)	<0.001	<0.001	0.282
Serum biochemistry	LDL-C (mmol/L)	<0.001	<0.001	0.102
Serum biochemistry	LDH (U/L)	<0.001	<0.001	0.060
Serum biochemistry	GGT (U/L)	<0.001	<0.001	0.183
Antioxidant parameters	T-AOC (U/mL)	<0.001	<0.001	<0.001
Antioxidant parameters	T-SOD (U/mL)	<0.001	<0.001	<0.001
Antioxidant parameters	POD (U/mL)	<0.001	<0.001	0.113
Antioxidant parameters	MDA (nmol/mL)	<0.001	<0.001	<0.001
Antioxidant parameters	GRAC (unitless)	<0.001	<0.001	0.723
Antioxidant parameters	GSH-Px (U/mL)	<0.001	<0.001	<0.001
Antioxidant parameters	CAT (U/mL)	<0.001	<0.001	0.065

Table S3F. Continued.

Outcome category	Variable	*p*-treatment	*p*-linear	*p*-quadratic
Immune and inflammatory parameters	IgA (μg/mL)	<0.001	<0.001	0.142
Immune and inflammatory parameters	IgG (μg/mL)	<0.001	<0.001	0.218
Immune and inflammatory parameters	IgM (μg/mL)	0.006	0.011	0.607
Immune and inflammatory parameters	IL-1β (ng/L)	<0.001	<0.001	<0.001
Immune and inflammatory parameters	IL-10 (ng/L)	<0.001	<0.001	0.004
Immune and inflammatory parameters	Hs-CRP (mg/L)	<0.001	0.020	<0.001
Immune and inflammatory parameters	LZM (μg/L)	0.389	0.165	0.971
Immune and inflammatory parameters	TNF-α (pg/mL)	<0.001	<0.001	0.191
Immune and inflammatory parameters	IL-6 (pg/mL)	<0.001	<0.001	0.101
Serum indicators related to intestinal permeability	DAO (U/mL)	<0.001	<0.001	0.408
Serum indicators related to intestinal permeability	D-lactate (mmol/L)	<0.001	<0.001	0.860
Gene expression	*PTGS2* (COX-2) relative expression	<0.001	<0.001	0.361
Gene expression	*SOD1* relative expression	0.685	0.366	0.553
Gene expression	*HMOX1* (HO-1) relative expression	<0.001	<0.001	0.066
Gene expression	*NFE2L2* (Nrf2) relative expression	<0.001	<0.001	0.001
Gene expression	*NQO1* relative expression	<0.001	<0.001	0.264

p-treatment was obtained from one-way ANOVA; *p*-linear and *p*-quadratic were obtained from orthogonal polynomial contrasts. For diarrhea rate, the reported *p*-values were obtained from analyses of arcsine square-root-transformed proportions; untransformed percentages are presented in Table S3A.

Table S4. Analytical endpoints, measurement methods, and reported units.

Endpoint	Manufacturer	Catalog No.	Measurement method	Reported unit
Serum biochemistry				
ALB (albumin)	Nanjing Jiancheng	A028-1-1	Bromocresol green colorimetry	g/L
TP (total protein)	Nanjing Jiancheng	A045-2-2	Coomassie brilliant blue method	g/L
GLU (glucose)	Nanjing Jiancheng	A154-1-1	Glucose oxidase method	mmol/L
UN (urea nitrogen)	Nanjing Jiancheng	C013-1-1	Diacetyl monoxime colorimetry	mmol/L
TBIL (total bilirubin)	Nanjing Jiancheng	C019-1-1	Chemical oxidation method (microplate)	μmol/L
DBIL (direct bilirubin)	Nanjing Jiancheng	C019-2-1	Chemical oxidation method (microplate)	μmol/L
ALP (alkaline phosphatase)	Nanjing Jiancheng	A059-1-1	Visible-light colorimetry	King units/100 mL
AST (aspartate aminotransferase)	Nanjing Jiancheng	C010-2-1	Reitman-Frankel method (microplate)	U/L
ALT (alanine aminotransferase)	Nanjing Jiancheng	C009-2-1	Reitman-Frankel method (microplate)	U/L
CK (creatine kinase)	Nanjing Jiancheng	A032-1-1	Colorimetry	U/mL
CREA (creatinine)	Nanjing Jiancheng	C011-1-1	Picric acid method (microplate)	μmol/L
TG (triglycerides)	Nanjing Jiancheng	A110-1-1	GPO-PAP method	mmol/L
TC (total cholesterol)	Nanjing Jiancheng	A111-1-1	COD-PAP method	mmol/L
HDL-C (HDL cholesterol)	Nanjing Jiancheng	A112-1-1	Direct method (catalase method)	mmol/L
LDL-C (LDL cholesterol)	Nanjing Jiancheng	A113-1-1	Direct method (surfactant clearance method)	mmol/L
LDH (lactate dehydrogenase)	Nanjing Jiancheng	A020-1-1	Colorimetry	U/L
GGT (γ-glutamyl transferase)	Nanjing Jiancheng	C017-1-1	Colorimetry	U/L
Antioxidant status				
T-AOC (total antioxidant capacity)	Nanjing Jiancheng	A015-1-1	Colorimetry	U/mL
T-SOD (total superoxide dismutase)	Nanjing Jiancheng	A001-2-1	Hydroxylamine method	U/mL
POD (peroxidase)	Nanjing Jiancheng	A084-1-1	Colorimetry	U/mL
MDA (malondialdehyde)	Nanjing Jiancheng	A003-2-1	Thiobarbituric acid method	nmol/mL
GRAC (glutathione reductase activity coefficient)	Nanjing Jiancheng	A104-1-1	Colorimetric glutathione reductase activation coefficient assay	unitless
GSH-Px (glutathione peroxidase)	Nanjing Jiancheng	A005-1-2	Colorimetry	U/mL
CAT (catalase)	Nanjing Jiancheng	A007-2-1	Ultraviolet method	U/mL

Table S4. Continued.

Endpoint	Manufacturer	Catalog No.	Measurement method	Reported unit
Immune and inflammatory markers				
IgA (immunoglobulin A)	Nanjing Jiancheng	H108-1-2	ELISA	μg/mL
IgG (immunoglobulin G)	Nanjing Jiancheng	E026-1-1	Immunoturbidimetry	μg/mL
IgM (immunoglobulin M)	Nanjing Jiancheng	H109-1-1	ELISA	μg/mL
IL-1β (interleukin-1β)	Nanjing Jiancheng	H002-1-1	ELISA	ng/L
IL-10 (interleukin-10)	Nanjing Jiancheng	H009-1-2	ELISA	ng/L
TNF-α (tumor necrosis factor-α)	Nanjing Jiancheng	H052-2-2	ELISA	pg/mL
IL-6 (interleukin-6)	Nanjing Jiancheng	H007-1-1	ELISA	pg/mL
Hs-CRP (high-sensitivity C-reactive protein)	Nanjing Jiancheng	E024-1-1	Latex-enhanced immunoturbidimetry	mg/L
LZM (lysozyme)	Nanjing Jiancheng	A050-1-1	Turbidimetry	μg/L
Serum intestinal permeability-related markers				
DAO (diamine oxidase)	Nanjing Jiancheng	A088-1-1	Ultraviolet colorimetry	U/mL
D-lactate	Nanjing Jiancheng	A019-3-1	Colorimetry	mmol/L
Whole-blood gene expression				
PTGS2 (COX-2), *SOD1*, *HMOX1* (HO-1), *NFE2L2* (Nrf2), and *NQO1*	—	—	RT-qPCR; *ACTB* reference gene; $2^{-\Delta\Delta Ct}$ method	Relative expression

All commercial assay kits were obtained from Nanjing Jiancheng Bioengineering Institute (Nanjing, China) and used according to the manufacturers' instructions. For ELISA-based markers (IgA, IgM, IL-1β, IL-10, TNF-α, and IL-6), the reported values are concentrations derived from the corresponding standard curves. *ACTB* served as the reference gene for RT-qPCR. ALP activity is reported in King units/100 mL, as specified by the manufacturer's kit instructions.
